# Identification of a synergistic interaction between endothelial cells and retinal pigment epithelium

**DOI:** 10.1111/jcmm.13175

**Published:** 2017-04-12

**Authors:** Carrie Spencer, Stephanie Abend, Kevin J. McHugh, Magali Saint‐Geniez

**Affiliations:** ^1^ Schepens Eye Research Institute Mass. Eye and Ear Boston MA USA; ^2^ Department of Biomedical Engineering Boston University Boston MA USA; ^3^ Department of Ophthalmology Harvard Medical School Boston MA USA

**Keywords:** retinal pigment epithelium, endothelial cells, differentiation, matrix deposition, angiogenesis, heterotypic cell interactions

## Abstract

The retinal pigment epithelium located between the neurosensory retina and the choroidal vasculature is critical for the function and maintenance of both the photoreceptors and underlying capillary endothelium. While the trophic role of retinal pigment epithelium on choroidal endothelial cells is well recognized, the existence of a reciprocal regulatory function of endothelial cells on retinal pigment epithelium cells remained to be fully characterized. Using a physiological long‐term co‐culture system, we determined the effect of retinal pigment epithelium‐endothelial cell heterotypic interactions on cell survival, behaviour and matrix deposition. Human retinal pigment epithelium and endothelial cells were cultured on opposite sides of polyester transwells for up to 4 weeks in low serum conditions. Cell viability was quantified using a trypan blue assay. Cellular morphology was evaluated by H&E staining, S.E.M. and immunohistochemistry. Retinal pigment epithelium phagocytic function was examined using a fluorescent bead assay. Gene expression analysis was performed on both retinal pigment epithelium and endothelial cells by quantitative PCR. Quantification of extracellular matrix deposition was performed on decellularized transwells stained for collagen IV, fibronectin and fibrillin. Our results showed that presence of endothelial cells significantly improves retinal pigment epithelium maturation and function as indicated by the induction of visual cycle‐associated genes, accumulation of a Bruch's membrane‐like matrix and increase in retinal pigment epithelium phagocytic activity. Co‐culture conditions led to increased expression of anti‐angiogenic growth factors and receptors in both retinal pigment epithelium and endothelial cells compared to monoculture. Tube‐formation assays confirmed that co‐culture with retinal pigment epithelium significantly decreased the angiogenic phenotype of endothelial cells. These findings provide evidence of critical interdependent interactions between retinal pigment epithelium and endothelial cell involved in the maintenance of retinal homeostasis.

## Introduction

Located between the photoreceptor cells and the choroidal vasculature, the retinal pigment epithelium (RPE) is a polarized monolayer of pigmented epithelial cells, which forms outer blood–ocular barrier 1. The RPE, which sits on the collagen and elastin rich Bruch's membrane (BrM), performs several functions that are essential to maintain normal retinal physiology and visual function including light‐energy adsorption, ion and water transport, immunological barrier formation, visual product recycling, phagocytosis, and secretion of growth factors and cytokines 1. Consequently, RPE defects and/or atrophy secondary to ageing, injury (traumatic or toxic), and diseases can lead to photoreceptor degeneration and vision loss 2.

In addition to support photoreceptor survival and visual function, the RPE also controls formation and maintenance of the choriocapillaris, a dense vascular network of fenestrated endothelial cells underlying the BrM. Clinical and experimental evidences have indicated that the developmental formation of the choroidal vasculature depends on proper RPE differentiation 3, 4, 5. This morphogenic effect appears to depend mainly on the polarized secretion by the RPE of vascular endothelial growth factor A (VEGFA) as transgenic animals with RPE‐specific deletion of VEGFA display major developmental defects of the outer‐retina including the lack of choriocapillaris 6. The crucial inter‐relationship between the RPE and the choroidal endothelial cell (EC) is not limited to development and persists in adult. Histopathological evaluation of the atrophic form of age‐related macular degeneration indicates that early changes in the RPE layer precede the atrophy of the choriocapillaris, suggesting that choriocapillaris loss is secondary to RPE dysfunction 7, 8, 9. The observations of an age‐dependent loss of choriocapillaris fenestration and integrity in animal models of RPE atrophy 10, 11 and in mice lacking the soluble forms of VEGFA 12 further support this concept.

While the trophic role of RPE on the adjacent choroidal ECs is obvious, the existence of reciprocal instructive functions of the choriocapillaris on the RPE remains elusive. Choroidal atrophy and/or ischaemia is clinically and experimentally associated with regional RPE lesions and serous detachment 13, 14. Reduced vascularity *in vivo* is associated with unavoidable confounding factors such as loss of oxygen, nutrients and circulating factors that will alter cell function and survival of the surrounding tissues preventing any potential conclusion on a direct effect of EC loss on the RPE. However, the recent discovery that endothelial cells exert important morphogenic activities through the release of tissue‐specific angiocrine factors 15 suggests that EC in the outer‐retina complex could exert similar instructive cues that remain to be characterized.

Because normal interaction between RPE and ECs appears critical for the maintenance of the outer‐retina structure and function, pathological alterations of this relationship could be the primary event leading to vision‐threatening pathologies such as macular degeneration. A better characterization of the homeostatic RPE‐EC relationships is therefore particularly significant. Prior *in vitro* studies aimed at characterizing such interactions have focused on RPE‐dependent functions in pathological processes, mainly choroidal neovascularization (CNV) and epithelial–mesenchymal transition (EMT), using disease‐mimicking conditions such as hypoxia 16, 17, 18, 19, direct cell contact 17, subconfluent or activated RPEs in high serum 18, 20. While these models provided important insights on the cellular and molecular regulation of EC angiogenic status, the identification of the role of RPE‐EC heterotypic interactions on the formation and maintenance of the outer‐retinal complex requires the use of a more physiological model such as the one described in our study in which human RPE and EC were co‐cultured under quiescent conditions and their interdependent functions directly evaluated.

## Materials and methods

### Cell culture

The human retinal pigment epithelial cell line (ARPE‐19, CRL‐2302) was obtained from American Type Culture Collection (Manassas, VA, USA). ARPE‐19 cells were grown in DMEM/F‐12 (Lonza, Walkersville, MD, USA) supplemented with 10% foetal bovine serum (FBS; Atlanta Biologicals, Lawrenceville, GA, USA), 1% Glutamax (Lonza) and 1% penicillin–streptomycin (Lonza) at 37°C, 10% CO_2_. Primary human umbilical vein endothelial cells (HUVECs) were kindly provided by Ms. Case (Center for Excellence in Vascular Biology, Brigham and Women's Hospital, Boston, MA, USA). HUVECs from passages 3 through 6 were cultured in plastic flasks coated with 0.1% gelatin (Sigma‐Aldrich, St. Louis, MO, USA), in EGM‐2 (Lonza) with 20% FBS, SingleQuots supplements (Lonza), 1% glutamax and 1% penicillin–streptomycin at 37°C, 10% CO_2_.

### Co‐culture conditions

For all conditions, polyester transwell inserts for six‐ or 12‐well plates with 0.4‐μm pores (Corning Life Sciences, Corning, NY, USA) were coated with laminin (Sigma‐Aldrich) on the topside, for the ARPE‐19, and/or gelatin on the bottom side, for the HUVECs. For co‐cultures, HUVECs (6.0 × 10^4^ cells/cm^2^) were first seeded on the bottom side of transwells placed upside down inside a large Petri dish and incubated for 4–6 hrs in a CO_2_ incubator to allow for cell adhesion. The transwells were then placed right side up into the appropriate well‐plate, and complete EGM‐2 media with 20% serum was added to the bottom well (HUVEC side). ARPE‐19 cells were then plated at confluence (1.7 × 10^5^ cells/cm^2^) on the topside in DMEM/F12, 10% serum. In some cases, HUVECs were plated on the gelatin‐coated bottom of the well containing the transwell insert, instead of directly on the underside of the transwell. Twenty‐four hours after plating, the culture media was replaced by EBM‐2 (Lonza) with 1% FBS, 1% glutamax and 1% penicillin–streptomycin. Cells were maintained in this low serum co‐culture media for up to 4 weeks. Plating density and use of low serum were specifically selected to allow for maturation and polarization of the ARPE‐19 21. Cell viability was quantified by adding 100 μl of 0.4% trypan blue dye to 100 μl of cell suspension for up to 5 min. and counting the numbers of stained (dead) and unstained (live) cells on a haemocytometer. For haematoxylin and eosin (H&E) staining, cells attached to the transwells were briefly fixed in methanol (Sigma‐Aldrich), stained with a HARLECO Hemacolor stain set (EMD Millipore, Billerica, MA, USA). To allow for proper visualization of the EC, RPE cells on the opposite side of the membrane were carefully removed manually with sterile cotton swabs (Dynarex, Orangeburg, NY, USA) and PVA surgical spears (Eye Shield Technology Houston, TX, USA). The transwells were air‐dried, and the membranes removed from the insert with a fine scalpel blade (Miltex, York, PA; no. 11). The membranes were mounted with the HUVEC side up on slides with a xylene‐based mounting medium (Electron Microscopy Sciences, Hatfield, PA, USA) and imaged using a Axioskop 2 mot plus (Carl Zeiss Microscopy, Thornwood, NY, USA) running AxioVision 4.8 software (Carl Zeiss Microscopy).

### Scanning electron microscopy

Cells on transwells were fixed in a solution of 0.1 M sodium cacodylate, 0.1 M sucrose, and 3% glutaraldehyde (Electron Microscopy Sciences) for 24 hrs. The samples were then dehydrated by sequential incubation in 35, 50, 70, 95 and two 100% ethanol for 10 min. each. Samples were then covered with hexamethyldisilazane (HMDS), which was subsequently allowed to evaporate in a chemical fume hood at room temperature. Transwells were then detached from their supports, mounted on aluminium stubs, sputtered with 10 nm gold‐palladium alloy using a Cressington 108 Auto Sputter Coater (Watford, UK) and imaged using a Zeiss *Supra* 55 VP Field Emission Scanning Electron Microscope (Carl Zeiss Microscopy) at 5.0 kV acceleration voltage.

### Gene expression analysis

Total RNA was isolated from cells using RNA‐Bee solution (Iso‐Tex Diagnostics Inc., Pearland, TX, USA). Quantitative RT‐PCR was performed, first by amplifying the cDNA using the Bio‐Rad iScript cDNA Synthesis Kit (Hercules, CA, USA) according to the kit instructions. Then, the expression of genes associated with RPE differentiation, function and matrix deposition was analysed by quantitative PCR (qPCR) using a Roche LightCycler480 system, with the FastStart SYBR Green Master Mix (Roche, Basel, Switzerland) according to the manufacturer's instructions. The primer sequences used are listed in the Table 1. Relative gene expression was determined using the delta‐delta Ct method and normalized with the housekeeping genes GAPDH and HPRT1 to increase quantification accuracy 22.

**Table 1:** List of human genes studies and primer sequences used for qPCR analysis

Gene name (symbol)	Forward primer	Reverse primer
Collagen 4A4 (COL4A4)	5′‐AGAGATTGCTCTGTTTGCCAC‐3′	5′‐CGGTCCCCTCTCATTCCTT‐3′
Cellular retinaldehyde‐binding protein (RLBP1)	5′‐GCTGCTGGAGAATGAGGAAACT‐3′	5′‐TGAACCGGGCTGGGAAGGAATC‐3′
Basic fibroblast growth factor (FGF2)	5′‐GCGACCCACACGTCAAACTA‐3′	5′‐TCCCTTGATAGACACAACTCCTC‐3′
Glyceraldehyde 3‐phosphate dehydrogenase(GAPDH)	5′‐CCCATCACCATCTTCCAGGA‐3′	5′‐CATCGCCCCACTTGATTTTG‐3′
Hypoxanthine‐guanine phosphoribosyltransferase (HPRT)	5′‐TCAGTCAACGGGGGACATAAA‐3′	5′‐GGGGCTGTACTGCTTAACCAG‐3′
Matrix metallopeptidase 2 (MMP2)	5′‐CTTCCAAGTCTGGAGCGATGT‐3′	5′‐TACCGTCAAAGGGGTATCCAT‐3′
Myosin VIIa (MYO7A)	5′‐CATGACGGGGAGTCCACAG‐3′	5′‐TCTCTTGCTAGGTTGACAGAGG‐3′
Na+/K+ ATPase (ATPA1)	5′‐ACAGCCTTCTTCGTCAGTATCGT‐3′	5′‐CGAATTCCTCCTGGTCTTACAGA‐3′
Nuclear factor‐like 2 (NFE2L2 or NRF2)	5′‐CTTTTGGCGCAGACATTCCC‐3′	5′‐GACTGGGCTCTCGATGTGAC‐3′
Occludin (OCLN)	5′‐CCCTTTTAGGAGGTAGTGTAGGC‐3′	5′‐CCGTAGCCATAGCCATAACCA‐3′
Orthodenticle homeobox 2 (OTX2)	5′‐TAAGCAACCGCCTTACG‐3′	5′‐GCACTTAGCTCTTCGATT‐3′
Pigment epithelium‐derived factor (SERPINF1 or PEDF)	5′‐TATCACCTTAACCAGCCTTTCATC‐3′	5′‐GGGTCCAGAATCTTGCAATG‐3′
Superoxide dismutase 2 (SOD2)	5′‐CGTTCAGGTTGTTCACGTAGG‐3′	5′‐CCTCACATCAACGCGCAGAT‐3′
Tropoelastin (ELN)	5′‐AGTCGCAGGTGTCCCTAGTG‐3′	5′‐ACCAGCACCAACTCCAAGTC‐3′
Vascular endothelial growth factor A (VEGFA)	5′‐GGGCAGAATCATCACGAAGTG‐3′	5′‐ATTGGATGGCAGTAGCTGCG‐3′
Tight junction protein 1 (TJP1 or ZO1)	5′‐CAACATACAGTGACGCTTCACA‐3′	5′‐GACGTTTCCCCACTCTGAAAA‐3′

Validated primers for collagen 1A1 (*COL1A1*), collagen 18A1 (*COL18A1*), decorin (*DCN*), fibronectin 1 (*FN1*), laminin (*LAMB2*), lysyl oxidase (*LOX*), nerve growth factor (NGF), retinal pigment epithelium‐specific protein (*RPE65*), thrombospondin‐1 (*THBS1*) and TIMP metallopeptidase inhibitor 3 (*TIMP3*) were obtained from SABiosciences.

### VEGF secretion

Cell culture media conditioned for 72 hrs was collected from the apical and basal chambers. Concentration of secreted vascular endothelial growth factor A (VEGF‐A) was measured using human VEGF‐A ELISA kit (R&D Systems, Minneapolis, MN, USA).

### Histology and immunohistochemistry

ARPE‐19 cells on transwell membranes were fixed in 4% paraformaldehyde (Electron Microscopy Sciences) for 10 min. Samples were permeabilized for 5 min. with 0.01% Triton X‐100 (Sigma‐Aldrich) and blocked in 1% bovine serum albumin (BSA; Sigma‐Aldrich) and 3% goat serum (Sigma‐Aldrich) for 1 hr at room temperature. Samples were incubated overnight at 4°C in blocking buffer with anti‐ZO‐1 (Invitrogen, Carlsbad, CA, USA; 1:100). The following day, samples were washed 3 times for 10 min. in phosphate‐buffered saline (PBS; Sigma‐Aldrich) and incubated for 2 hrs at room temperature with blocking buffer with a Daylight 549 goat anti‐rabbit secondary antibody (Jackson Immunoresearch Laboratories, West Grove, PA, USA; 1:300), and DAPI (Invitrogen). Images were taken with an Axioskop 2 mot plus microscope (Carl Zeiss Microscopy).

### Quantification of extracellular matrix protein deposition

Transwell inserts were collected at 2 weeks for extracellular matrix (ECM) deposition analysis. Transwells were washed with PBS and then incubated in 0.02 M NH_4_OH (Sigma‐Aldrich) for 5–10 min. to remove adherent cells. Transwell membranes were fixed for 15 min. in cold 4% paraformaldehyde and blocked for 45 min. in PBS with 2% FBS and 0.5% BSA at room temperature. The samples were incubated in the primary antibodies, either collagen IV (Sigma‐Aldrich; 1:1000), fibronectin (Abcam, Cambridge, MA, USA; 1:50) or fibrillin (EMD Millipore; 1:100), for two hours at room temperature. The samples were washed in PBS with 0.3% Triton X‐100 and then incubated with the appropriate secondary antibody, either Alexafluor 594 Rabbit anti‐Mouse (Invitrogen; 1:100) or Alexafluor 488 Goat anti‐Mouse (Invitrogen; 1:100) for one hour at room temperature. The samples were imaged using an Axioskop 2 mot plus microscope. Ten fields per transwell were imaged using a 40×/0.75 NA Plan‐Neofluar objective and identical exposure settings. The images were analysed using Photoshop CS6 (Adobe, San Jose, CA, USA). Median fluorescence intensity (MFI) area coverage was quantified for each image and averaged per transwell.

### Phagocytosis assay

ARPE‐19 phagocytic function was measured using a fluorescence bead test 23. FITC‐conjugated 0.7μm latex beads (Sigma‐Aldrich) were diluted to a concentration of 160 beads/cell in medium, applied to the apical side of the ARPE‐19 cells and incubated for 18 hrs. The cells were then washed thoroughly with PBS, fixed 5 min. at room temperature with 4% paraformaldehyde and viewed by epifluorescence microscopy using a Zeiss Axioskop 2. Six fields representing a total area of 3.54 mm^2^ were recorded for each transwells. Fluorescence intensity corresponding to the phagocytized/bound beads was quantified by pixel densitometry using Adobe Photoshop CS6 software (Adobe) and expressed as a per cent of monoculture control.

### Endothelial tube‐formation assay

Human umbilical vein endothelial cells were cultured in mono‐ and co‐culture conditions as previously described for 2 weeks. HUVECs were trypsinized from the bottom side of the transwells and the number of live cells quantified using a trypan blue viability assay. Cells were plated in 96‐well plate previously coated with 50 μl of Cultrex^®^ Basement Membrane Extract (BME; Trevigen, Gaithersburg, MD, USA) at a density of 15,000 live cells per well. Cells were incubated for 8 hrs in EGM2 media containing 20% serum and supplemented with 25 ng/ml of VEGF‐A. Tube‐like networks were imaged on an inverted microscope (Nikon, Tokyo, Japan) at a 4× magnification. Tube covered area, total tube length, number of branch points and loops were measured using the automated image analysis software Wimasis WimTube (Wimasis GmbH, Munich, Germany) 24.

### Statistical analysis

Values are expressed as mean ± S.E.M. and analysed by unpaired Student's *t*‐test using Prism 6 (Graphpad Software, La Jolla, CA, USA) unless stated otherwise (***P < 0.001, **P < 0.01, *P < 0.05, ns: *P* > 0.05).

## Results

### Long‐term trophic effect of RPE on ECs

**Figure 1:**
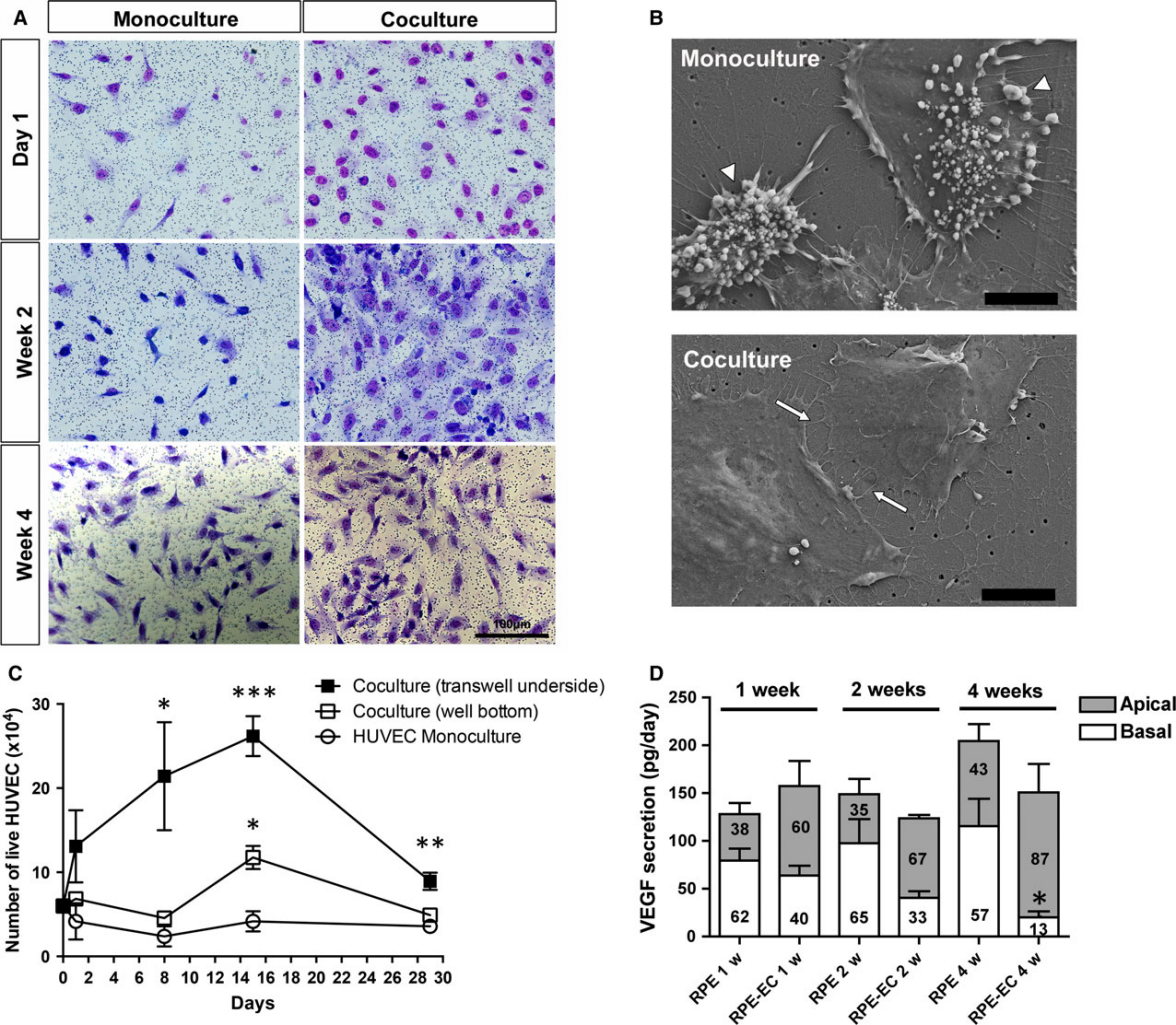
Trophic effect of RPE on EC survival and proliferation. (**A**) HUVEC density and morphology were analysed by H&E staining of culture transwells over a span of 4 weeks. (**B**) Scanning electron microscope images of HUVECs on culture transwells for 2 weeks. Cells in monoculture demonstrated extensive shrinking and blebbing (arrowheads) characteristic of apoptosis. In co‐culture condition, HUVECs displayed a normal flatten morphology and extended arrays of filopodia, many of them interconnecting (arrows). (**C**) HUVECs viability was measured using a trypan blue exclusion assay. In absence of serum and trophic cytokines, monocultured HUVECs died rapidly while cells in co‐cultures are maintained and even proliferate for the first 2 weeks when seeded on the opposite side of the RPE (*n* = 3 per time‐point, anova followed by Tukey's multiple comparison test ***P < 0.001, **P < 0.01, *P < 0.05). (**D**) VEGF‐A secretion was measured over a span of 4 weeks from both the apical (ARPE‐19 side) and the basal (HUVEC side) of the transwells. While total VEGF secretion is similar in both mono‐ and co‐culture, the basal concentration of VEGF‐A was strongly reduced in co‐culture conditions (*n* = 3 per time‐points, *P < 0.05). Scale bar is 100 μm in A and 10 μm in B. ECs: endothelial cells; HUVEC: human umbilical vein endothelial cells; RPE: retinal pigment epithelium.

We developed an *in vitro* system where RPE and endothelial cells were co‐cultured under conditions replicating the physiological and anatomical relationship between this two cell types. Culture conditions were primarily assessed by analysing the morphology and viability of primary human ECs (HUVECs) co‐cultured with ARPE‐19 cells. In monoculture, HUVECs survival requires high serum and growth factors (Fig. S1). However, HUVEC proliferation and survival could be achieved in low serum conditions for at least 2 weeks when co‐cultured with quiescent and confluent ARPE‐19 (Fig. 1A–C). Survival of ECs cultured with RPE was associated with improved morphology such as the loss of the extensive blebbing and shrinking characteristic of apoptotic cells 25 that was observed in monocultured HUVECs (Fig. 1B). While we found no evidence of diaphragmed fenestrae formation, numerous well‐defined pores and caveolae‐like vesicles were observed in co‐culture HUVECs, particularly in regions of highly attenuated cytoplasm (Fig. S2). Surprisingly, the trophic effect of RPE on EC survival was only observed when ECs were plated directly under the ARPE‐19 cells and not when ECs were plated on the bottom of the well (Fig. 1C), suggesting that proximity or direct cell–cell contact may be required. However, quantification of the number of endothelial processes able to extend through the entire length of the 0.4‐μm transwell pores (<3% of the pores, Fig. S3) suggests that direct intercellular contact is uncommon. Thus, the trophic effect observed likely depends on local concentration of RPE‐derived growth factors such as VEGF‐A more than juxtacrine signalling. Based on these observations, all following co‐culture studies were conducted with HUVECs seeded on the underside of the transwells. To support the hypothesis that EC survival in our co‐culture system depends on RPE‐secreted angiogenic factor, the concentration of VEGF‐A present in conditioned medium was determined (Fig. 1D). As previously shown 26, 27, ARPE‐19 secreted a significant amount of soluble VEGF‐A preferentially directed towards the basal side (Fig. 1D). Interestingly, while the total concentration of VEGF‐A secreted by RPE in the medium was not significantly affected by culture conditions, lower levels were measured in the bottom well when in the presence of ECs, suggesting that soluble VEGF‐A released by RPE is utilized by ECs. No VEGF‐A was detected in conditioned media from HUVEC monocultures (data not shown).

### Effect of co‐culture on RPE matrix deposition

**Figure 2:**
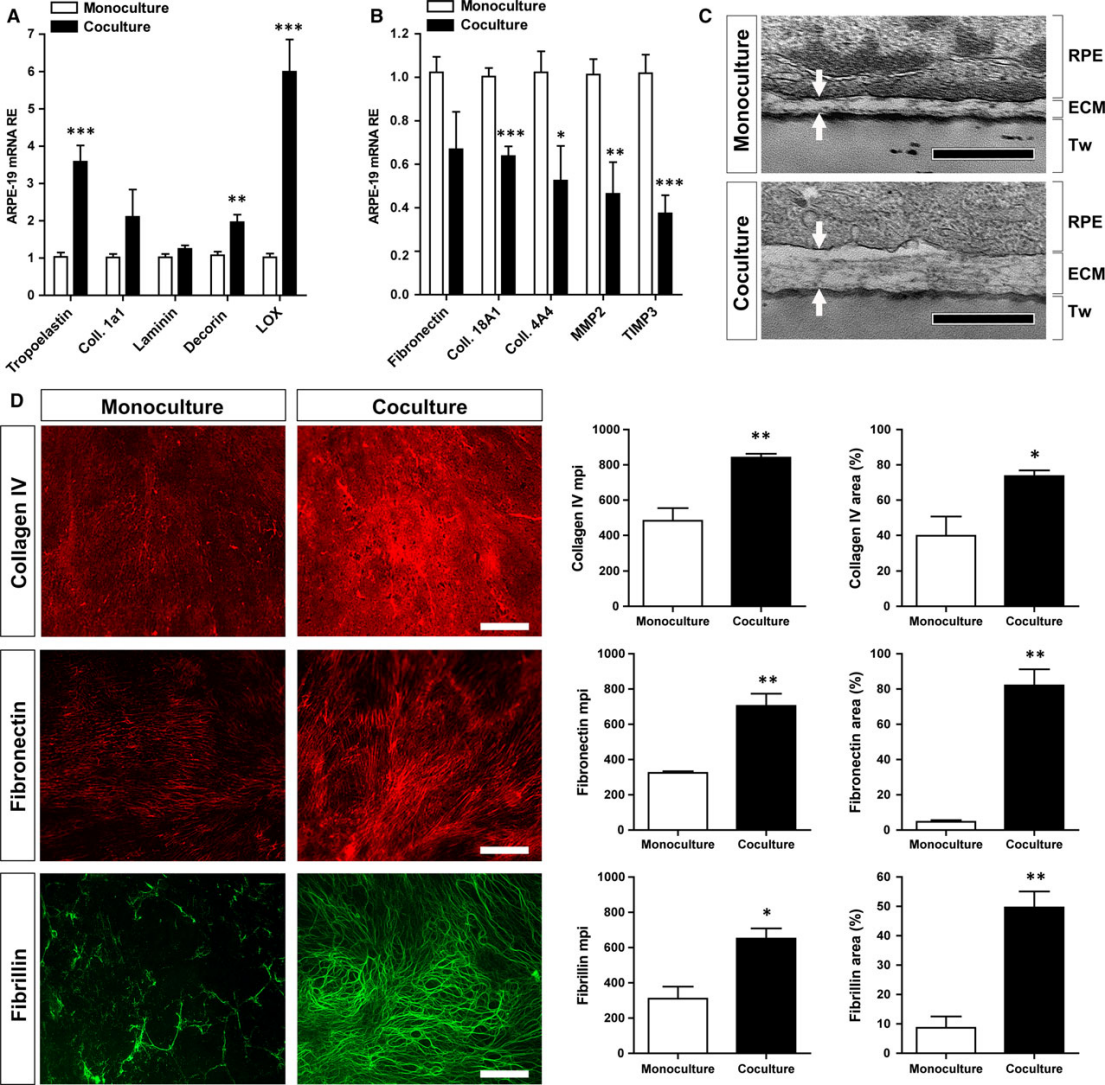
Effect of EC co‐culture on RPE matrix deposition. (**A‐B**) qPCR analysis of ECM‐related genes in ARPE‐19 cells cultured with or without EC for 2 weeks (*n* = 5–7 per conditions). (**A**) Gene expression analysis revealed increased expression of several matrix‐associated proteins, notably decorin, LOX and tropoelastin. (**B**) Several other proteins, however, including other types of collagen, MMP2 and TIMP3, were down‐regulated. (**C**) TEM analysis revealed an increase in extracellular matrix (ECM) deposits (defined by the two arrows) between the basal lamina of the ARPE‐19 (RPE) and the transwell (Tw) when the cells were in co‐culture with HUVECs for 4 weeks, as compared to ARPE‐19 cells in monoculture. (**D**) Immunofluorescent (IF) detection of collagen IV, fibronectin and fibrillin in the matrix‐network deposited by ARPE‐19 cells at 2 weeks. IF quantification using mean pixel intensity (mpi) and coverage area of images showed a significant increase in RPE extracellular matrix deposition in the co‐culture condition (*n* = 3 per conditions). Scale bar is 0.5 μm in C and 50 μm in D. ECM: extracellular matrix; ECs: endothelial cells; HUVEC: human umbilical vein endothelial cells; RPE: retinal pigment epithelium.

To determine whether the presence of ECs is able to increase extracellular matrix deposition by RPE, we first characterized the expression of matrix‐related genes in RPE grown in co‐ and monocultures. qPCR analysis revealed that the presence of ECs significantly increased the expression of tropoelastin (*ELN*), the proteoglycan decorin (*DCN*) and the enzyme lysyl oxidase (*LOX*) by ARPE‐19 (Fig. 2A). Interestingly, *COL18A1*,* COL4A1*,* MMP2* and *TIMP3* were also strongly repressed under co‐culture conditions (Fig. 2B). Decorin and LOX have been shown to be critical for the assembly of collagen and elastin fibrils 28, 29; therefore, we expected significant effect of the co‐culture condition on matrix deposition and organization This hypothesis was confirmed by TEM analysis that revealed a considerable increase in ECM deposited by RPE when co‐cultured with ECs (Fig. 2C). Deposition of collagen IV, fibronectin and fibrillin on the basal side of RPE was evaluated by immunofluorescence staining. Co‐culture conditions were associated with a significant improvement in the RPE‐deposited matrix as shown by increased pixel intensity and area coverage (Fig. 2D).

### Effect of ECs on RPE gene expression and functions

**Figure 3:**
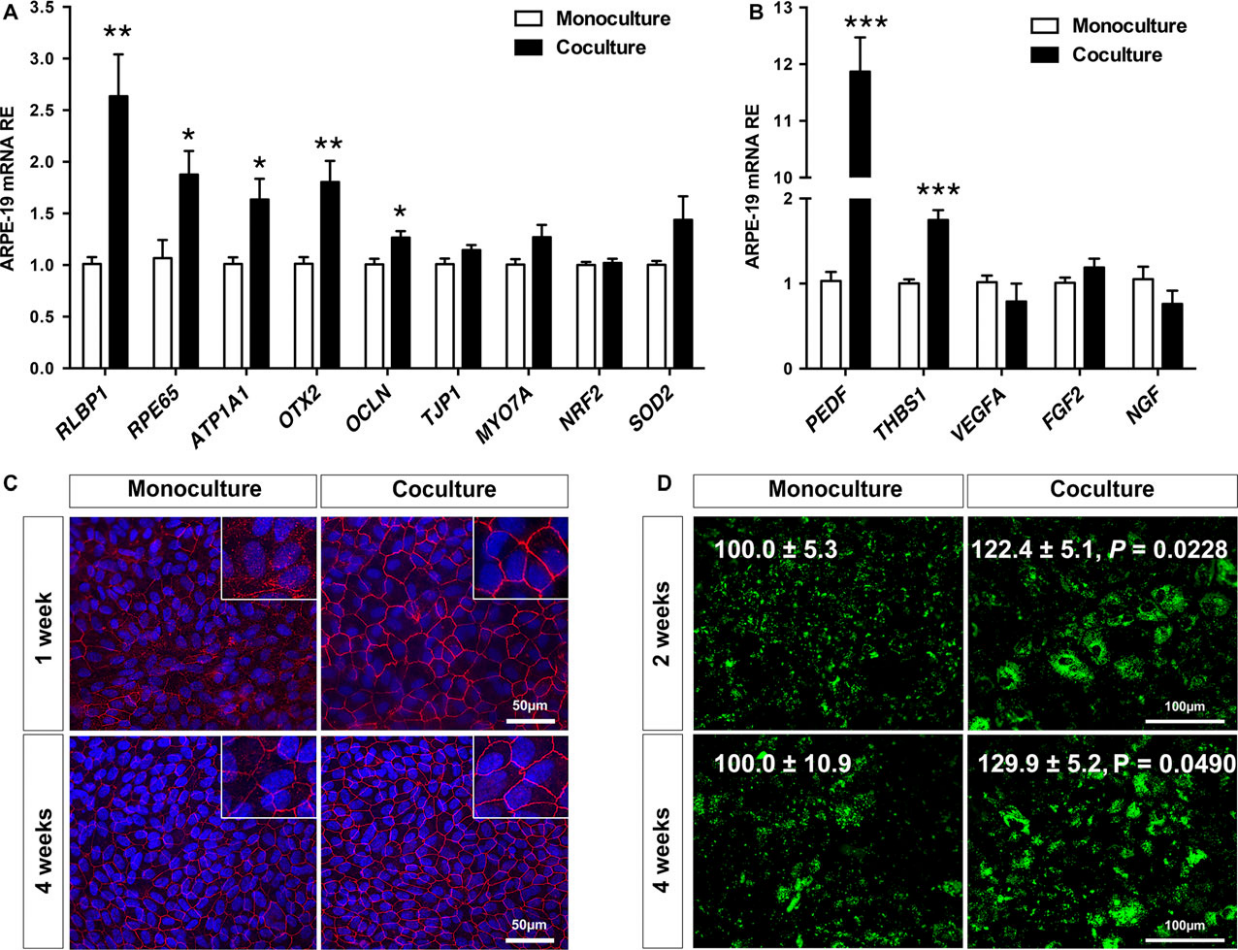
Effect of EC co‐culture on RPE‐specific gene expression and phagocytic activity. (**A‐B**) qPCR analysis of ARPE‐19 cells cultured with or without EC for 2 weeks (*n* = 5‐7, ***P < 0.001, **P < 0.01, *P < 0.05). (**A**) Gene expression analysis of RPE‐specific genes revealed that the presence of ECs (black bars) increases significantly the expression of numerous genes involved in visual function. (**B**) Gene expression analysis of RPE‐secreted growth factors revealed the significant induction of the neurotrophic factor PEDF and the anti‐inflammatory and anti‐angiogenic factor thrombospondin‐1 (THBS‐1). (**C**) Tight junction formation was evaluated by ZO‐1 IF on ARPE‐19 cultured with or without HUVECs for 1 and 4 weeks. Insets showing high magnification views demonstrate increased ZO‐1 staining intensity and improved membranous localization in co‐cultured RPE at both time‐points. (**D**) Micrographs of FITC‐labelled beads bound or internalized by ARPE‐19 cultured for 2 and 4 weeks with or without ECs. Quantification of beads uptake on *n* = 4 experimental repeats and expressed as percentile of monoculture controls is added to each panel. ECs: endothelial cells; HUVEC: human umbilical vein endothelial cells; RPE: retinal pigment epithelium.

The development of a long‐term co‐culture system of RPE and ECs allowed us to determine the effect of ECs on RPE behaviour and function. Gene expression analysis of ARPE‐19 cells cultured for 2 weeks in mono‐ and co‐culture conditions revealed that critical genes associated with RPE differentiation, homeostasis and function such as *RLBP1*,* RPE65*,* ATP1A1* **(**Na+/K + ‐ATPase) and *OTX2* were significantly up‐regulated in presence of ECs (Fig. 3A). Interestingly, co‐culture conditions were also associated with a specific induction of the neurotrophic factor *SERPINF1* (PEDF) and anti‐inflammatory factor *THBS1* (thrombospondin‐1) while other RPE‐secreted growth factors such as *VEGFA*,* FGF2* and *NFG* were not differentially expressed (Fig. 3B).

We then analysed the effect of HUVECs on ARPE‐19 monolayer organization and phagocytosis. Tight junction formation was evaluated by ZO‐1 immunofluorescence staining on ARPE‐19 culture with or without HUVECs for up to 4 weeks. As previously reported 21, 27, tight junction formation in ARPE‐19 is a gradual process and after 1 week of maturation, cells in monoculture displayed a moderate amount of ZO‐1 localization at the cell membrane and evidence of diffuse cytoplasmic staining. Interestingly, improved ZO‐1 localization at the cell membrane was observed in co‐culture conditions at all time‐points analysed (Fig. 3C). Phagocytosis of shed photoreceptor outer‐segments is one of the main functions of the RPE and is critical to maintain photoreceptor excitability. Phagocytic activity was evaluated by adding FITC‐labelled beads to the top of the culture chamber 23. ARPE‐19 cells in co‐culture conditions for 2 and 4 weeks showed a significant increase in phagocytic activity compared to cells in monoculture (Fig. 3D). This effect is not likely due to a change in microvilli density and/or morphology because no significant differences were observed in the two culture conditions by SEM (Fig. S4).

### Effect of RPE on EC angiogenic status

**Figure 4:**
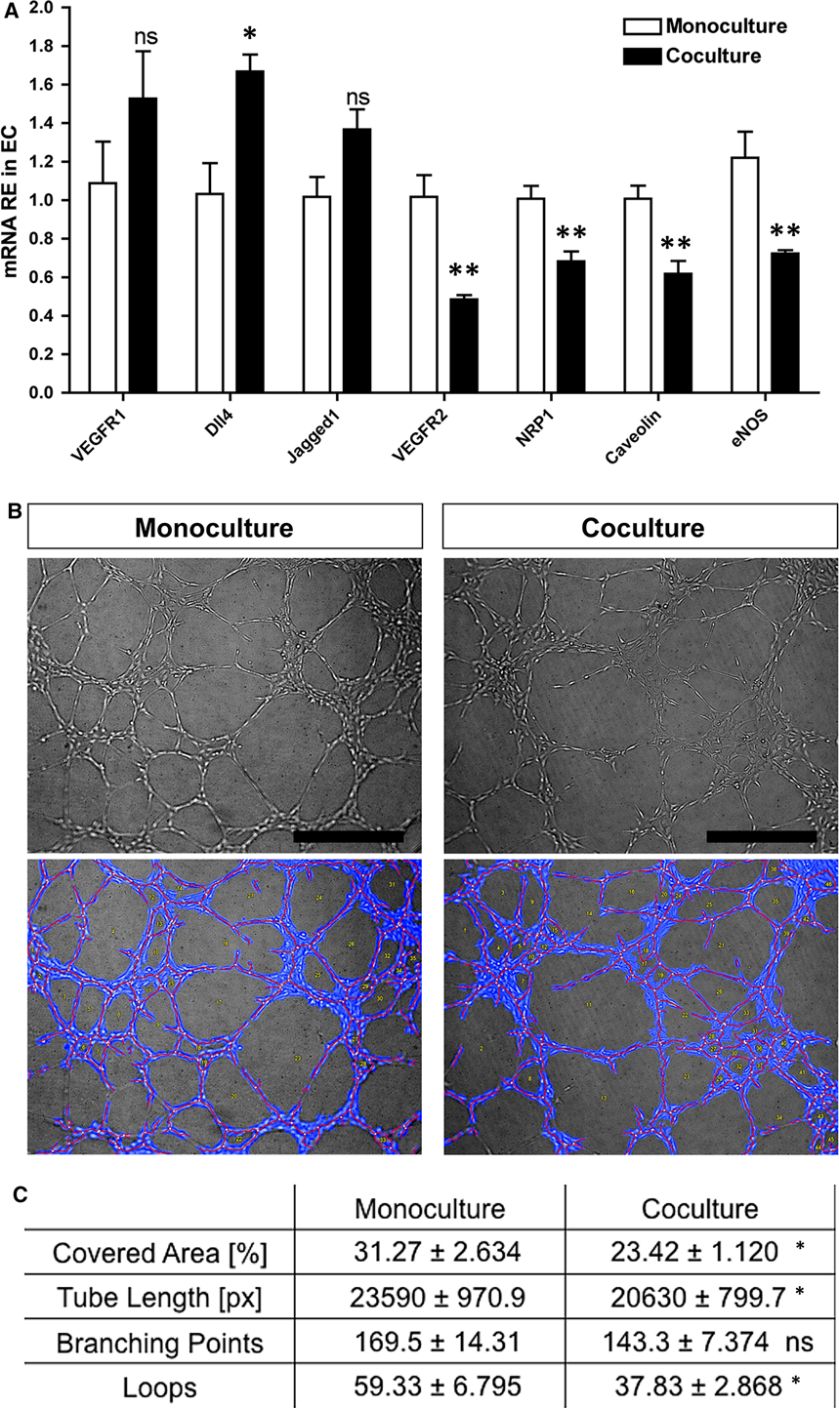
Induction of an anti‐angiogenic phenotype in EC co‐cultured with RPE. (**A**) qPCR analysis of angiogenesis‐related genes in HUVECs cultured with or without RPE for 2 weeks (*n* = 4 = , ***P < 0.001, **P < 0.01, *P < 0.05). (**B**) Following 2 weeks of co‐ or monoculture in low serum, HUVECs were collected and live cells were seeded at same density on BME gels in presence of high serum and 25 ng/ml of VEGF‐A. Capillary‐like tubes were imaged 8 hrs later using an inverted microscope (top panel) and analysed by automated quantitative analysis (lower panel shows processed images by Wimasis). (**C**) Results of tube characteristics quantification (*n* = 7, mean ± S.D., *P < 0.05). Scale bar is 500 μm. ECs: endothelial cells; HUVEC: human umbilical vein endothelial cells; RPE: retinal pigment epithelium.

The two secreted factors that were the most prominently induced in RPE by co‐culture conditions, PEDF and THBS1, are both characterized by their strong anti‐angiogenic activity suggesting that presence of RPE may not only improve EC survival but also maintain their quiescent state. To test this hypothesis, we evaluated the angiogenic phenotype of HUVECs by gene expression analysis and tube‐formation assay. Interestingly, co‐culture with RPE led to the significant up‐regulation of the Notch1 ligand *DLL4* and down‐regulation of multiple pro‐angiogenic proteins including VEGFR2, NRP1 and eNOS, suggesting that HUVECs cultured with RPE acquire an anti‐angiogenic phenotype (Fig. 4A). To confirm this observation, we collected HUVECs after 2 weeks of mono‐ and co‐culture and conducted an *in vitro* tube‐formation assay to determine their angiogenic capacity. Analysis of the capillary‐like structures formed under highly pro‐angiogenic conditions (high serum and presence of VEGF‐A) obviously demonstrated that co‐culture with RPE inhibits the pro‐angiogenic response of ECs (Fig. 4B and C).

## Discussion

Biomimetic *in vitro* models of the outer‐retina are critical for the detailed characterization of the cellular and molecular processes involved under normal and pathological conditions. While optimal organotypic models of the RPE‐BrM‐choroidal EC complex should recapitulate the physiological organization and phenotypic characteristics of the human outer‐retina as closely as possible, the experimental study constraints must also be taken into consideration. Here, the selection of epithelial (ARPE‐19) and endothelial cell (HUVEC) sources was motivated by the rigorous requirements needed to reveal the interdependent relationship between the two cell types. Although primary foetal human RPE (hfRPE) displays superior pigmentation, barrier function and retinol metabolism compared to ARPE‐19 30, their maintenance requires the use of a medium rich in serum (5%) and adjuvants including the N1 neurobasal growth supplement, taurine and triiodothyronine hormone 31 whose presence in the co‐culture medium would negatively alter the homeostatic and quiescent status of the endothelial cells. Similarly, the use of primary human choroidal endothelial cells in our model was impeded by limited access to donors and large intervariability. Moreover, there is substantial evidence that *in situ* tissue specificities of EC, such as fenestrations and vascular markers, are lost when cells are isolated and expanded in culture 32, 33 likely due to the deprivation from micro‐environmental cues and from the influence of parenchymal cells 34. HUVECs were selected because of their high reproducibility, purity, extensive characterization and phenotypic plasticity allowing them to differentiate upon specific culture conditions 35. Survival of human primary ECs such as HUVECs in monoculture condition requires high serum and additional growth factors. However, long‐term HUVECs maintenance could be achieved in low serum condition when co‐cultured with ARPE‐19, indicating that RPE cells are able to support EC survival. This trophic effect of RPE on ECs appeared to depend on the close proximity of cells because there was less survival observed when ECs where seeded on the bottom well than on the underside of the transwell. Because the transwell's pore diameter (0.4 μm) and thickness (10 μm) do not allow for prominent direct cell contact (Fig. S3), this heterotypic trophic effect likely depends on local concentration of vasculo‐trophic growth factors such as VEGF‐A secreted by the RPE. It is also conceivable that the presence of RPE could stimulate the formation of long filopodia by the EC allowing for juxtacrine signalling. However, careful analysis of TEM pictographs from co‐culture transwells did not reveal the presence of EC processes able to extend through the membrane pores and to form direct contact with the RPE membrane. Therefore, the RPE‐EC interrelation determined in our study is likely dependent on paracrine signalling.

Our findings indicate that presence of EC robustly promotes RPE maturation and functions as demonstrated by improved tight junction formation, increased expression of RPE‐specific genes and enhanced phagocytic activity. RPE functions and morphology are intimately regulated by its interactions with the BrM. Interestingly, we observed that presence of EC in close proximity to RPE modulates the expression of ECM components by the epithelial cells and enhances the deposition of a complex BrM‐like matrix mainly *via* the induction of decorin and LOX, two essential fibrillogenesis regulators. This is in agreement with animal and clinical studies indicating that BrM formation and turnover is regulated by the choroidal ECs 36. Indeed, impaired choroidal development secondary to RPE‐specific VEGF deletion leads to the severe BrM attenuation to a single basal laminar layer 6. Similarly, clinical observations from patients with choroideremia, an X‐linked genetic disease, show that early degeneration of the choriocapillaris is associated with abnormal BrM organization 37. Alternatively, EC may directly regulate RPE maturation *via* the release of inductive and homeostatic paracrine factors. Microvascular EC‐derived angiocrine factors have recently shown to regulate the differentiation, regeneration and survival of numerous tissue and organs, including neural stem cells 38, liver 39, lung epithelium 40. It is therefore possible that the organization and maintenance of the outer‐retina complex is similarly dependent of the cross‐interactions between RPE‐ and ECs‐derived paracrine factors that remain to be fully characterized.

One particularly intriguing observation in our study was the increased expression of the RPE‐derived anti‐angiogenic factors, PEDF and THBS1, concomitant with the suppression of EC pro‐angiogenic phenotype. Both PEDF and THBS1 are prominently localized in the RPE‐BrM complex where they are thought to prevent aberrant vessels growth into the subretinal space under physiological conditions. The decreased expression of these two endogenous angiogenic regulators observed in AMD patients 41 may therefore promote pathological choroidal neovascularization. Decorin, which we found to be induced in co‐cultured RPE, has also been shown to exert potent angiostatic activities 42 through its ability to inhibiting receptor tyrosine kinases such as IGFR1 43 and VEGFR2 44. Using a co‐culture system similar to ours, Du *et al*. 20 demonstrated that decorin overexpression in ARPE‐19 suppresses hypoxia‐induced VEGFA and inhibits the angiogenic activity of chorioretinal endothelial cells. Similarly to our results, RPE‐derived VEGFA levels were not affected by exogenous decorin under normoxic conditions 20. All together, our findings strongly support previous evidence that normal RPE is required for the maintenance of the choroidal vessels quiescence 41, 45, 46.

In conclusion, our study demonstrates the existence of a synergistic heterotypic relation between RPE and EC that improves RPE differentiation and deposition of a BrM‐like matrix while inhibiting the ECs angiogenic potential and emphasizes the importance of RPE‐EC interactions in the maintenance of retinal homeostasis. To further characterize the intercellular relationships between choroidal EC and RPE, *in vitro* systems combining freshly isolated primary or iPS‐derived cells 47, 48 under defined culture media with endogenous or bioengineered matrices known to improve cellular behaviour and phenotype 35, 49, 50, 51 should be investigated.

## Conflict of interest

The authors confirm that there is no conflict of interest associated with this publication.

## Supporting information


**Fig. S1** Effect of serum and growth factor supplements on HUVEC proliferation and survival.
**Fig. S2** SEM analysis of HUVEC in mono‐ and co‐culture conditions.
**Fig. S3** Quantification of EC processes extending across the culture transwell.
**Fig. S4** Apical microvilli formation in mono‐ and co‐cultured RPE.
