# Supplementary material for: Identification of a synergistic interaction between endothelial cells and retinal pigment epithelium
Source: J Cell Mol Med. 2017 Apr 12;21(10):2542–52. doi: 10.1111/jcmm.13175 (PMC5618686; doi:10.1111/jcmm.13175)
Supplement: Supplementary file 1 — Fig. S1 Effect of serum and growth factor supplements on HUVEC proliferation and survival. Fig. S2 SEM analysis of HUVEC in mono‐ and co‐culture conditions. Fig. S3 Quantification of EC processes extending across the culture transwell. Fig. S4 Apical microvilli formation in mono‐ and co‐cultured RPE. [file JCMM-21-2542-s001.pdf]

Supplemental information

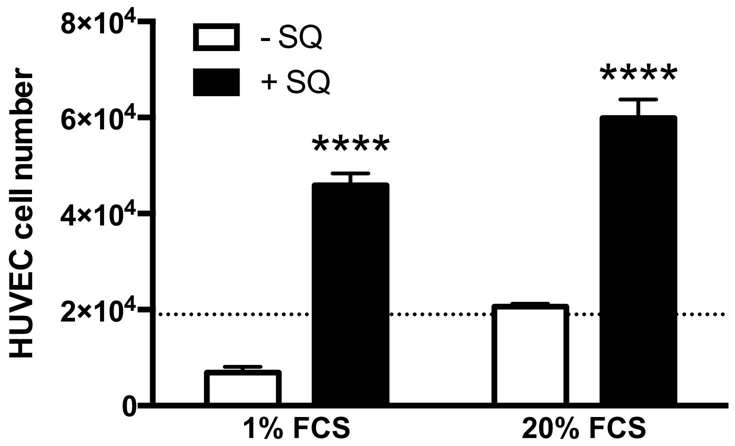

Supplemental Figure 1: Effect of serum and growth factor supplements on HUVEC proliferation and survival.

HUVECs were plated at 20,000 cells/cm^2 and cultured in EGM-2 medium supplemented with either 20% or 1% FBS and the EGM-2 Bulletkit (SQ) containing a panel of EC specific growth factors such as VEGF-A and IGF-1.The number of live cells was then analyzed by MTT assay 7 days later. Proliferation of ECs is only observed in medium containing the supplemental trophic growth factors (SQ) (black bars) and is relatively independent from the serum concentration which appears to control cells survival in the absence of supplemental factors (white bar). The dotted line represents the cell number at day 0, n=4.

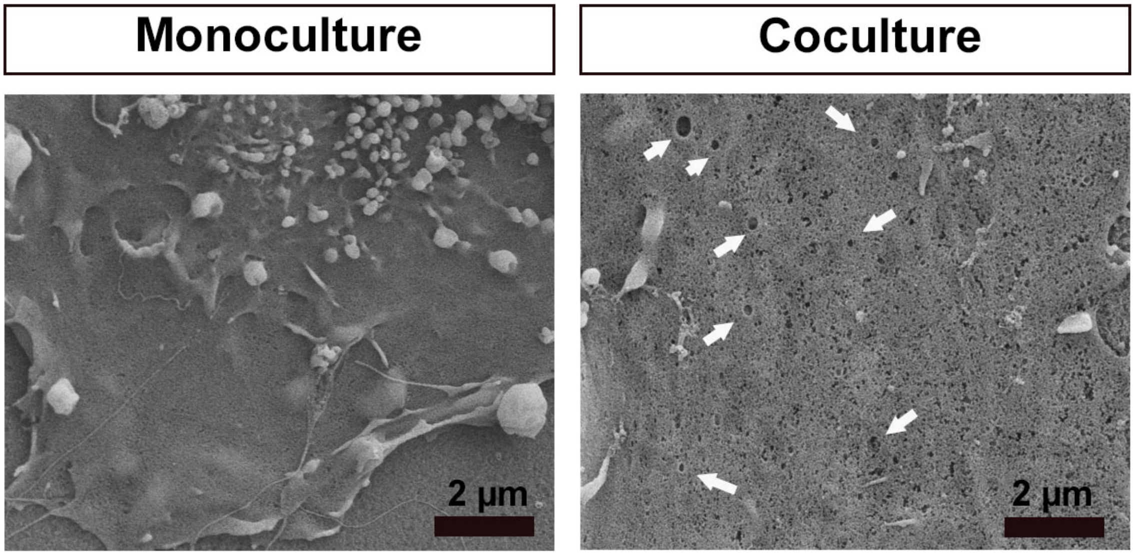

Supplemental Figure 2: SEM analysis of HUVEC in mono- and co-culture conditions.

Formation of pores and caveolae-like vesicles (arrows) were prominently observed in co-cultured HUVECs but not in monoculture conditions.

A

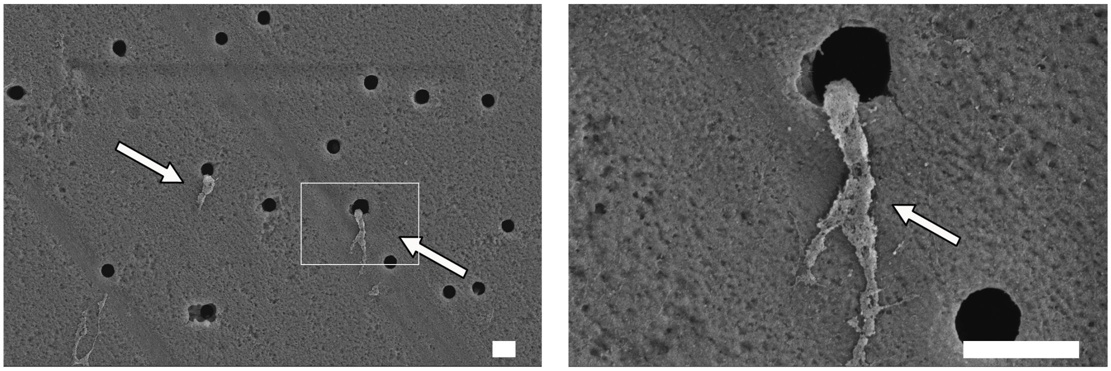

B

	# pores	# cellular processes	% pores w/ processes
Sample #1	236	7	2.97
Sample #2	265	3	1.13
Sample #3	240	7	2.91
Average ± SEM	247 ± 16	5 ± 2	2.33 ± 1.05

Supplemental Figure 3: Quantification of EC processes extending across the culture transwell.

HUVECs were plated at 60,000 cells/cm^2 on the bottom-side of a gelatin-coated culture transwells and maintained in 1% serum co-culture media for two weeks. (A) SEM of the opposite side of the transwell showing an example of EC filopodia extending through the transwell pores. (B) Quantification of the number of pores involved with endothelial processes. Scale bar is 1 μm.

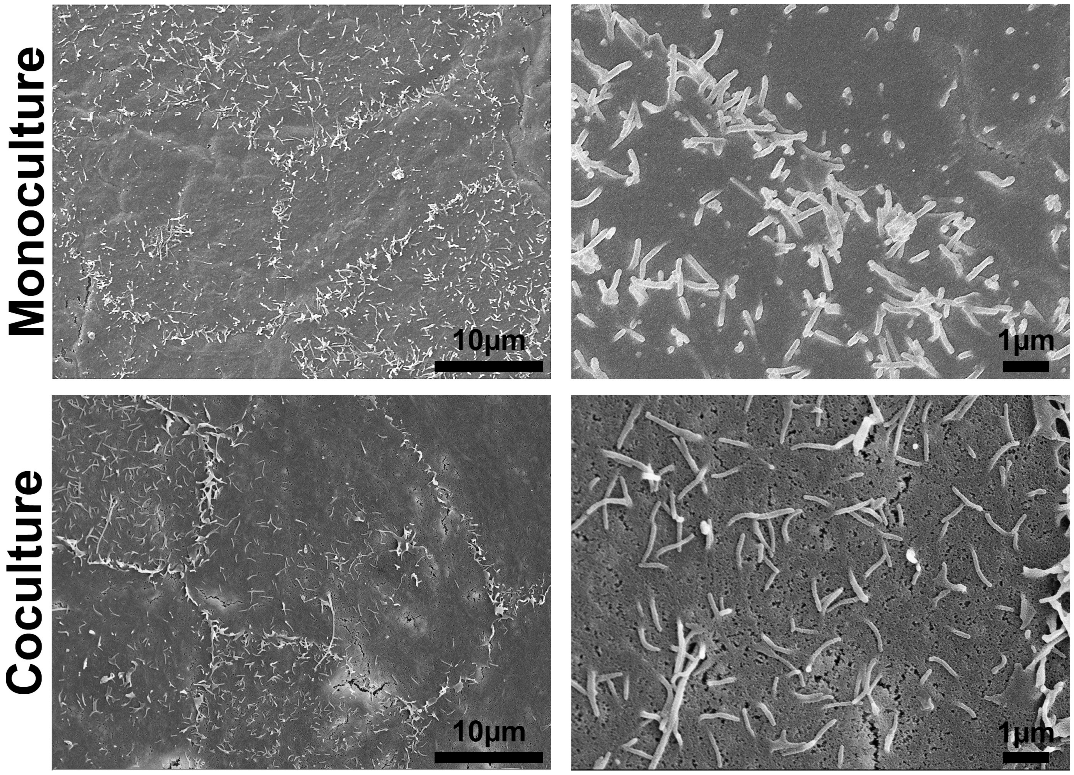

Supplemental Figure 4: Apical microvilli formation in mono- and co-cultured RPE. SEM micrograph of ARPE-19 cells differentiated for four weeks in mono- (top) and co-culture (bottom) conditions. Low (left panel) and high magnification (right panel) shows similar distribution and length of apical microvilli. Scale bar is 10 μm (left) and 1 μm (right).
